# Supplementary figures and images for: Simulated sunlight decreases the viability of SARS-CoV-2 in mucus
Source: PLoS One. 2021 Jun 10;16(6):e0253068. doi: 10.1371/journal.pone.0253068 (PMC8191973; doi:10.1371/journal.pone.0253068)

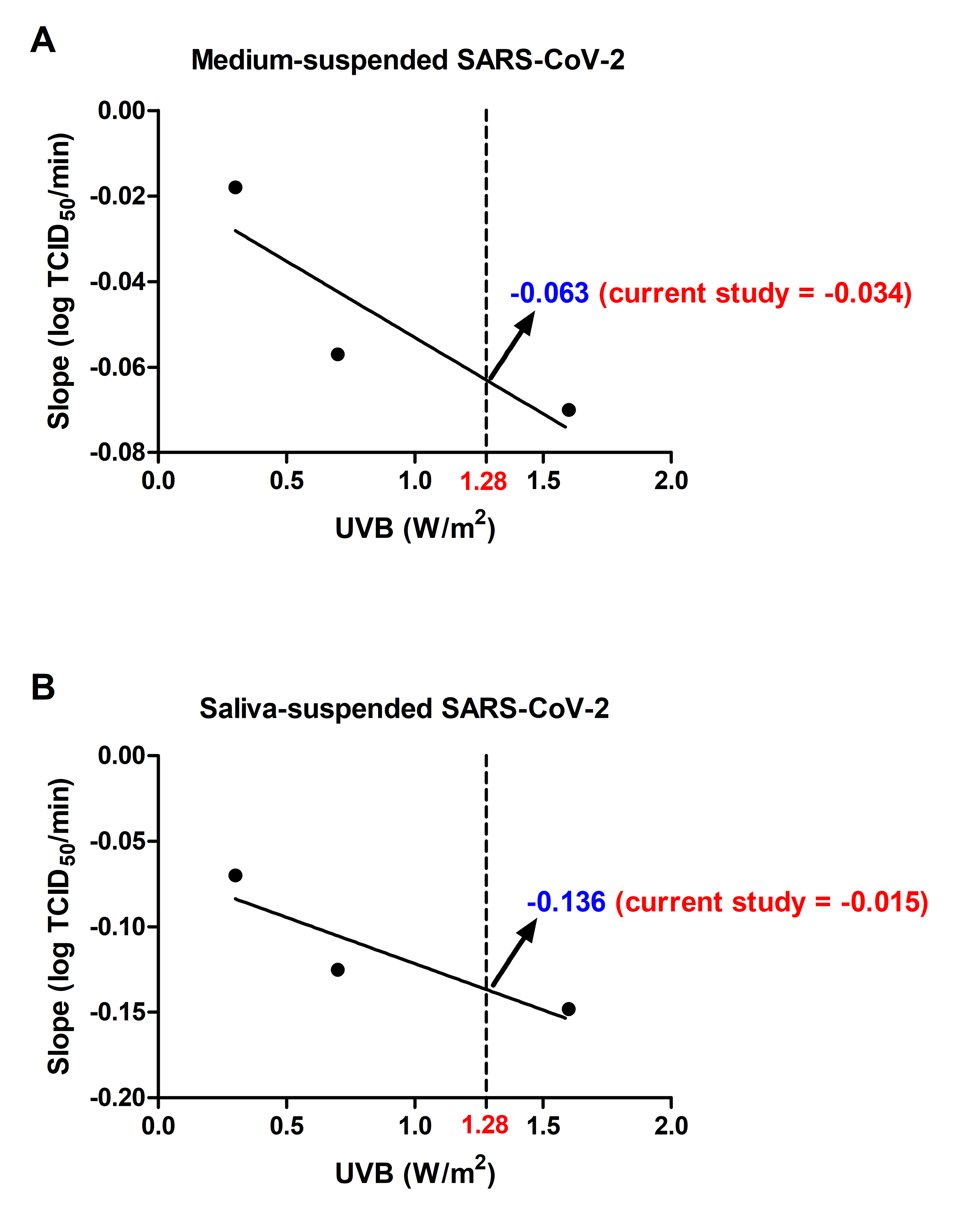

Supplement: S1 Fig — By plotting the viral decay rates of (A) medium- and (B) saliva-suspended SARS-CoV-2 observed by Ratnesar-Shumate et al. [22] at different UVB intensities, expected decay rates for medium- and mucus-suspended SARS-CoV-2 were extrapolated for the UVB intensity examined in the current study. In line with Ratnesar-Shumate et al.’s findings, the viral decay of medium-suspended virus (A) at a UVB intensity of 1.28 watts per square meter (W/m2) should have been -0.063 log10 TCID50/min; however, our results show it occurred at -0.034 log10 TCID50/min. Comparing bodily matrices (B), according to Ratnesar-Shumate et al., saliva-suspended SARS-CoV-2 should decay at a rate of -0.136 log10 TCID50/min when UVB equals 1.28 W/m2. The current study shows that mucus-suspended SARS-CoV-2 decays at a rate of over nine times slower, at 0.015 log10 TCID50/min. (TIF) [file pone.0253068.s001.tif]
