# Supplementary material for: Simulated sunlight decreases the viability of SARS-CoV-2 in mucus
Source: PLoS One. 2021 Jun 10;16(6):e0253068. doi: 10.1371/journal.pone.0253068 (PMC8191973; doi:10.1371/journal.pone.0253068)
Supplement: S1 Table — (DOCX) [file pone.0253068.s002.docx]

**S1 Table. Log_10_ TCID_50_/mL values used to create linear regression lines for each data set.**

| **Minutes post-desiccation** | **Titer of re-suspended virus (log10 TCID_50_/mL)** | | | | | |
| --- | --- | --- | --- | --- | --- | --- |
|  | **Sunlight** | | | **Control** | | |
|  | **Rep 1** | **Rep 2** | **Rep 3** | **Rep 1** | **Rep 2** | **Rep 3** |
| **Medium, controlled heat** | |  |  |  |  |  |
| 0 | 2.50 | 3.50 | 3.25 | 2.50 | 3.50 | 3.25 |
| 15 | 2.50 | 2.50 | 3.25 | 3.25 | 2.75 | 3.75 |
| 30 | 1.50 | 2.25 | 1.50 | 3.50 | 2.75 | 3.50 |
| 60 | 0.00 | 0.75 | 0.75 | 4.25 | 3.50 | 2.75 |
| 120 | 0.75 | 0.75 | 0.00 | 3.25 | 3.25 | 3.00 |
| **Medium, variable heat** | |  |  |  |  |  |
| 0 | 4.25 | 3.75 | 3.75 | 4.25 | 3.75 | 3.75 |
| 15 | 2.75 | 2.75 | 3.50 | 3.50 | 3.75 | 4.25 |
| 30 | 2.50 | 2.50 | 2.75 | 3.50 | 3.50 | 4.25 |
| 60 | 1.75 | 1.50 | 2.00 | 3.75 | 3.25 | 3.75 |
| 120 | 0.75 | 0.75 | 0.75 | 3.25 | 3.00 | 3.00 |
| **Mucus, controlled heat** | |  |  |  |  |  |
| 0 | 3.75 | 3.50 | 3.50 | 3.75 | 3.50 | 3.50 |
| 15 | 2.67 | 2.50 | 3.00 | 4.00 | 3.50 | 4.75 |
| 30 | 2.50 | 2.25 | 2.75 | 3.75 | 3.50 | 3.50 |
| 60 | 0.75 | 0.75 | 2.50 | 4.50 | 4.00 | 3.67 |
| 120 | 2.25 | 1.75 | 1.75 | 3.75 | 3.25 | 3.50 |
| 180 | 0.75 | 0.75 | 0.75 | 3.46 | 4.00 | 3.25 |
| 240 | 0.75 | 0.75 | 0.75 | 2.75 | 3.50 | 2.75 |
| **Mucus, variable heat** | |  |  |  |  |  |
| 0 | 3.75 | 3.50 | 3.50 | 3.75 | 3.50 | 3.50 |
| 15 | 2.50 | 2.50 | 2.75 | 3.50 | 2.75 | 3.50 |
| 30 | 2.50 | 2.46 | 2.50 | 2.50 | 3.25 | 3.50 |
| 60 | 1.75 | 0.75 | 0.75 | 3.25 | 2.75 | 3.50 |
| 120 | 1.75 | 0.75 | 0.75 | 2.75 | 2.75 | 2.75 |
| 180 | 0.75 | 0.75 | 0.75 | 3.25 | 3.25 | 2.75 |
| 240 | 0.75 | 0.75 | 0.00 | 3.25 | 2.50 | 2.75 |

TCID_50_/mL, 50% tissue culture infectious dose per milliliter. Blue font denotes values under the limit of quantitation that were excluded from graphing and statistical analyses.
