# Supplementary material for: Simulated sunlight decreases the viability of SARS-CoV-2 in mucus
Source: PLoS One. 2021 Jun 10;16(6):e0253068. doi: 10.1371/journal.pone.0253068 (PMC8191973; doi:10.1371/journal.pone.0253068)
Supplement: S2 Table — (DOCX) [file pone.0253068.s003.docx]

**S2 Table. Best fit and goodness of fit parameters for linear regression lines constructed for each data set.**

| **Parameter** | **Variable Heat** | | | | **Controlled Heat** | | | |
| --- | --- | --- | --- | --- | --- | --- | --- | --- |
|  | **Medium** | | **Mucus** | | **Medium** | | **Mucus** | |
|  | **Sun** | **Control** | **Sun** | **Control** | **Sun** | **Control** | **Sun** | **Control** |
| Y-intercept | 3.717 ± 0.142 | 3.937 ± 0.113 | 3.115 ± 0.163 | 3.307 ± 0.114 | 3.194 ± 0.245 | 3.225 ± 0.177 | 3.138 ± 0.144 | 3.891 ± 0.133 |
| Slope | -0.034 ±  0.004 | -0.007 ±  0.002 | -0.015 ± 0.004 | -0.002 ± 0.001 | -0.044 ± 0.013 | -0.001 ± 0.003 | -0.011 ± 0.002 | -0.003 ± 0.001 |
| 95% CI | -0.044 to  -0.025 | -0.011 to  -0.002 | -0.024 to  -0.007 | -0.004 to  -0.0003 | -0.074 to  -0.015 | -0.006 to  -0.007 | -0.016 to  -0.006 | -0.005 to  -0.001 |
| r^2^ | 0.874 | 0.461 | 0.654 | 0.229 | 0.003 | 0.003 | 0.674 | 0.280 |
| Sy.x | 0.318 | 0.294 | 0.412 | 0.353 | 0.470 | 0.470 | 0.377 | 0.411 |
| df | 10 | 12 | 9 | 19 | 7 | 13 | 11 | 19 |

CI, confidence interval; r^2^, coefficient of determination; Sy.x, standard error of estimate; df, degrees of freedom.
