# Supplementary material for: Simulated sunlight decreases the viability of SARS-CoV-2 in mucus
Source: PLoS One. 2021 Jun 10;16(6):e0253068. doi: 10.1371/journal.pone.0253068 (PMC8191973; doi:10.1371/journal.pone.0253068)
Supplement: S3 Table — (DOCX) [file pone.0253068.s004.docx]

**S3 Table. Results of D’Agostino-Pearson normality tests used to confirm Guassian residuals for each data set.**

| **Parameter** | **Variable Heat** | | | | **Controlled Heat** | | | |
| --- | --- | --- | --- | --- | --- | --- | --- | --- |
|  | **Medium** | | **Mucus** | | **Medium** | | **Mucus** | |
|  | **Sun** | **Control** | **Sun** | **Control** | **Sun** | **Control** | **Sun** | **Control** |
| D'Agostino & Pearson omnibus K^2^ statistic | 0.092 | 2.463 | 2.780 | 1.819 | 0.012 | 0.622 | 0.613 | 3.619 |
| p-value | 0.9552 | 0.2919 | 0.2490 | 0.4027 | 0.9938 | 0.7328 | 0.7360 | 0.1637 |
| Assumption of normality met? | Yes | Yes | Yes | Yes | Yes | Yes | Yes | Yes |
