# Supplementary material for: Simulated sunlight decreases the viability of SARS-CoV-2 in mucus
Source: PLoS One. 2021 Jun 10;16(6):e0253068. doi: 10.1371/journal.pone.0253068 (PMC8191973; doi:10.1371/journal.pone.0253068)
Supplement: S4 Table — (DOCX) [file pone.0253068.s005.docx]

**S4 Table. Results of runs tests used to confirm the assumption of linearity for each regression line.**

| **Parameter** | **Variable Heat** | | | | **Controlled Heat** | | | |
| --- | --- | --- | --- | --- | --- | --- | --- | --- |
|  | **Medium** | | **Mucus** | | **Medium** | | **Mucus** | |
|  | **Sun** | **Control** | **Sun** | **Control** | **Sun** | **Control** | **Sun** | **Control** |
| p-value | 0.6667 | 0.5000 | 0.5000 | 0.2000 | 1.0000 | 0.5000 | 0.5000 | 1.0000 |
| Assumption of linearity met? | Yes | Yes | Yes | Yes | Yes | Yes | Yes | Yes |
